# The opinions of ambulance personnel regarding using a heated mattress for patients being cared for in a cold climate – An intervention study in ambulance care

**DOI:** 10.1080/22423982.2017.1379305

**Published:** 2017-10-08

**Authors:** Jonas Aléx, Tom Uppstu, Britt-Inger Saveman

**Affiliations:** ^a^ Department of Nursing and affiliated with the Arctic Research Centre at Umeå University, Research fields: Prehospital emergency care, Umeå University, Umeå, Sweden; ^b^ Department of Nursing, Umeå University, Umeå, Sweden

**Keywords:** Thermal comfort, hypothermia, chilling, active warming, care suffering, ambulance care

## Abstract

The purpose of the study was to describe the opinions of ambulance personnel regarding differences between using a heated mattress and a standard ambulance mattress. This study was an intervention study with pre- and post-evaluation. Evaluations of the opinions of personnel regarding the standard unheated mattress were conducted initially. After the intervention with new heated mattresses, follow-up evaluations were conducted. Ambulance personnel (n=64) from an ambulance station in northern Sweden took part in the study, which ran from October 2014 until February 2016. There were differences in opinions regarding the standard unheated mattress and the new heated mattress. The evaluation of the proxy ratings by the personnel showed that the heated mattress was warmer than the standard mattress, more pleasant to lie on and that patients were happier and more relaxed than when the standard mattress was used. The ambulance personnel in this study rated the experience of working with the heated mattress as very positive and proxy rated that it had a good effect on patient comfort. A heated mattress can be recommended for patients in ambulance care, even if more research is needed to receive sufficient evidence.

## Background

Since the 1980s there has been rapid progress in ambulance care. A lot of new equipment has also appeared, for example, the defibrillator, CPAP and pulse oximeter [1]. On the other hand, it is still rare to find simple care measures for optimising the ambulance environment, which in sub-arctic areas often is cold in the winter months, e.g. ensuring that patients are not subjected to discomfort when they lie down on a cold ambulance mattress [2].

Patients are rarely able to influence their environment and the care provider should, therefore, create a balance between the environment and the medical treatment [3]. The study by Aléx *et al*. [4] is one of a few that highlights the importance of knowing when patients are cold and understanding their need for heat to achieve thermal comfort, because the ambulance environment is often too cold in winter. Keeping a normal indoor temperature inside the ambulance compartment in wintertime is difficult due to cold ambient temperatures and lack of active heating systems. Therefore, there is a need for additional active heat sources [5]. An ambient temperature of 27ᵒC is required for the body to be able to maintain normal body temperature without clothes at rest in conditions with no wind [6]. A study of thermal comfort showed that patients need an indoor temperature of 24ᵒC during winter time [7]. A study conducted during winter time in northern Sweden with 62 patients showed that 44% deemed the back of the ambulance to be cold and 85% of the patients had a finger temperature below 31ᵒC [8]. The body’s defence against chilling is shivering, which occurs at a temperature of 35ᵒC, and a chilled person who shivers can increase their heat production up to 5 times [9]. This can lead to oxygen consumption increasing up to 6 times, which in turn can lead to rapid tissue death due to lack of oxygen [10]. Shivering has been described as a very uncomfortable experience for chilled people, both pre-hospital and post-operative, but on provision of heat, for example the pain and anxiety diminish [11]. One way to reduce shivering in ambulance care is to have patients lie on a warm surface [2].

### Risk groups that are easily affected by chilling in ambulance care

Chilling is usually due to the person losing heat through, among other means, convection and conduction [6]. With conduction, heat is conducted away from one object to another via direct contact, such as between a patient and a stretcher mattress. With convection, heat is transferred from an object to the air or water, for example, a person’s body gives off heat to cooler air. Patients at a greater risk of chilling include the elderly, who have a reduced capacity to shiver; young children, who have a large skin surface area in relation to their body weight; people under the influence of alcohol or drugs; people being treated with, for example, β-blockers or antidepressants; and people who have suffered trauma [12]. Chilling and hypothermia affects the body’s coagulation system, so patients bleed more [13,14] and this can, therefore, be lethal in connection with trauma [15]. Drugs or alcohol can contribute to chilling due to physiological causes or due to a deterioration in judgement [16].

### Active warming as treatment

The use of active warming (from below and heated blankets) during surgical procedures resulted in less post-operative cold and shivering and fewer cases of hypothermia [17–19].

In an experiment, 23 healthy individuals who were exposed to cold (+2ᵒC) for 10 minutes were then asked, in an environment at room temperature (+21ᵒC), to lie on a standard ambulance mattress and on a heated ambulance mattress. The results show a difference between the two mattresses in the skin temperature on the back. The test subjects also perceived greater comfort when they lay on the heated mattress [2].

Active warming is more than twice as effective as reflective aluminium foil with regard to re-heating and is, therefore, probably useful in a pre-hospital environment [20]. Chemical heating pads placed on top of patients have been shown to be effective in reducing shivering and improving pre-hospital thermal comfort [21]. Patients given active warming in ambulances and in air ambulances and those given warm chemical heating pads during transport had a higher body temperature compared with patients given a heated drip or blankets [22]. Injured patients in a cold pre-hospital environment reported difficulties in relaxing because they were cold and shivering. The pain was made worse by being cold and the cold was described as worse than the pain, despite the fact that they were suffering injuries such as fractures. Those given active warming (heated pads) perceived that the heat spread through their whole body; it was pleasant and the shivering stopped; they felt less discomfort and they became calm. Those patients given passive warming needed additional warming [4]. Using active warming that is distributed from below provides a positive effect for thermal comfort [23]. Within this area more research is needed on sources of active warming through e.g. experimentation and interventions, leading to implementation in pre-hospital emergency care in order to minimise the consequences of cold and the discomfort patients are subjected to [4,24].

Changes of working procedures are often perceived as complicated processes that in turn give rise to frustration and powerlessness. There is also often a lack of support from management [25] and it has become apparent that it is important to have a manager who can cooperate and manage personnel in how changes should be implemented [26]. A new procedure should be functional in operation, function as expected and satisfy those involved [27]. It is, therefore, important to investigate what ambulance personnel perceive of using a heated mattress on the stretcher, compared with a standard stretcher mattress.

## Purpose

The purpose is to describe the opinions of ambulance personnel regarding differences between using a heated mattress and a standard ambulance mattress.

## Method

This is an intervention study with evaluations before and after the introduction of heated mattresses in ambulance care. The study was conducted over a period of 18 months in a cold climate at an ambulance station in northern Sweden.

### Intervention

This intervention study is a continuation of earlier research [2,4,5] and looks to enable implementation in ambulance care. The intervention consists of introducing a new type of heated mattress into ambulance operations during the months February to April 2015 and September to February 2016. The heated mattress runs off a 12 volt power supply provided by the ambulance. Power consumption is 50 watts and the mattress size is 50×150 cm^2^. The mattress is placed over the existing stretcher mattress. The mattress consists of conductive polymers, a plastic that conducts current and generates a temperature of 35ᵒC. Its surface is made of a plastic material. There is no exact data on how many patients were using the new heated mattresses during the trial period, but it is estimated to be around 1500 patients.

### Surveys 1 and 2

Survey 1 was created in October 2014, before the participants had access to the heated mattress. It consists of 12 statements on ambulance personnel’s opinions regarding the standard unheated mattress and use of extra blankets and covers and what they think of the patients’ experience of lying on the mattress. Participants evaluated each question on a visual analogue scale (100 mm) i.e. they placed a cross on the line, representing a figure between 0 and 100 millimetres. An example of a statement was “To what extent do you think that patients lying on the ambulance mattress are happy with it?” A cross to the far left was equivalent to 0 and “To a very low extent” and to the far right it was equivalent to 10 and “To a very great extent”. The survey contained statements on cleaning, on whether patients were considered to be happy and relaxed, and the extent to which the personnel made use of bedding.

Surveys 1 and 2 had comparable statements, apart from the fact that Survey 2 also contained two questions specifically directed at use of the heated mattress. Both surveys were pilot-tested before they were distributed to the participants.

### Procedure and data collection

The head of the ambulance service was contacted by telephone to be given verbal information about the intervention study and then consented to the heated mattresses being tested and evaluated by means of a survey. Survey 1 was distributed and completed during a training day for the staff. Before the evaluations using Survey 2, the head of the ambulance service was contacted again by telephone for verbal information and consent. Written information and a request for permission form were also sent out and thereafter signed. The procedure concerning information for participants regarding the follow-up evaluation in Survey 2 – and that participation is voluntary – was the same as for Survey 1. Survey 2 was distributed at morning meetings of personnel and the participants completed the survey at the meeting. New personnel (n=16) who had not taken part in or completed Survey 1 had to first complete Survey 1 and then Survey 2.

### Analysis

In both surveys the completed responses were measured on a line with an accuracy of a hundredth, i.e. 0–100 millimetres. The responses were entered into IBM’s SPSS software. Data was analysed using descriptive calculations, min–max, mean values and standard deviations. To analyse the differences between different groups, a t-test and chi-square test were used. A p-value of <0.05 was considered to show significant differences.

### Ethical considerations

The study followed the Helsinki declaration. An application for permission to conduct survey-based studies in the workplace was sent by the researchers to the department manager, who issued written permission to conduct the study. All participants in the study were told the purpose of the study and were informed that participation was voluntary, that they could withdraw their participation at any point and that responses would be submitted anonymously. As the study only included proxy ratings and did not interfere with patient care, no formal ethical review was performed.

## Results

Participants in Surveys 1 and 2 proved to be homogeneous in terms of gender, age and number of service years. Participants’ perceptions of their knowledge regarding the care of chilled patients increased during the investigation period. Background data is presented in Table 1.

**Table 1.** Participants’ sex, service years and estimated knowledge of care of chilled patients divided into those responding before (Survey 1) and those after the intervention (Survey 2).

	Survey 1	Survey 2	p-value
Number of participants (n)	64	50	
Sex (mean value), (%)			0.66
Male	49 (76.6)	36 (72)	
Female	15 (23.4)	14 (28)	
Service years (mean value)	11.4	11.3	0.97
Estimated knowledge*	59.3	66.3	0.01

* 0 = little knowledge; 100 = a lot of knowledge.

There was a significant difference between how the personnel evaluated the outcome of the heated mattress as opposed to the standard mattress. The heated mattress was evaluated as being warmer and more pleasant for the patients to lie on. The personnel also thought that the patients were happier and more relaxed compared with those lying on the standard mattress. The heated mattress was perceived as being easier to clean.

There is a trend, but no significant difference, showing that the extent of using extra bedding (winter bedding) was lower in Survey 2 and the personnel also estimated their ability to offer active warming to patients as higher compared with the estimates in Survey 1 (Table 2).

**Table 2.** Participants’ evaluation of Surveys 1 and 2 concerning access to active warming in the ambulance, if the mattress is cold or pleasant to sit on, if the patients are happy or relaxed, cleaning of the mattress and use of extra bedding (winter bedding).

	Survey 1				Survey 2				
	Min	Max	Mean	SD	Min	Max	Mean	SD	p-value
Access to active warming	1	98	20.8	24.4	1	100	34.2	26.8	0.07
Cold mattress	9	100	79.0	22.56	1	76	17.4	15.47	<0.001
Pleasant to sit on	1	92	17.9	19.8	49	100	84.1	12.23	<0.001
Happy patients	0	93	31.0	20.96	52	100	82.16	12.35	<0.001
Relaxed patients	1	94	26.92	20.0	43	100	75.33	16.83	<0.001
Cleaning*	0	99	34.5	27.26	0	88	15.94	18.15	<0.001
Winter bedding**	6	100	73.9	21.32	2	100	68.1	26.36	0.2

* Lower value = Easier to clean.

** Extra blanket underneath.

The specific questions regarding the heated mattress in Survey 2 showed that the heated mattress was not an extra burden in terms of connecting it to and disconnecting it from the power supply (m=24 millimetres), where 0 was to a very low extent and 100 to a very high extent. The personnel thought that there was increased comfort for patients who were cared for on a heated mattress in the ambulance (m=85 millimetres).

## Discussion

The main results of this study are that personnel evaluated the heated mattress compared to the standard mattress as being warmer and more pleasant for patients to lie on and that patients were rated happier and more relaxed. There is also a tendency for the heated mattress to have contributed to a reduction in the use of “winter bedding”, such as an extra blanket underneath.

The standard mattress was evaluated as colder than the other mattress. This can be explained by the fact that it assumes the same temperature as the ambient temperature compared with the heated mattress, which maintained a constant temperature of 35ᵒC. In an earlier Swedish study involving 62 patients in winter time, the standard ambulance mattress was on average −3.9ᵒC before loading into the ambulance, but at its coldest −22.3ᵒC [8]. The results of this study, together with the study by Aléx *et al*. [8], should be clear enough to support the implementation of heating mattresses. This may seem obvious, but it appears that findings need to be reported in research studies in order to be credible and meaningful for raising quality in pre-hospital care in a cold climate. In ambulance care it has long been recognised among the personnel that the standard ambulance mattress often is cold in winter. In a literature study it was noted that 40% of trauma patients with signs of hypoperfusion also displayed signs of hypothermia, resulting in poor treatment results and high mortality [28]. A meta-analysis showed that a reduction of approximately 1ᵒC in body temperature contributed to affected coagulation, with greater bleeding as a consequence [29]. It can be seen as an important measure to increase thermal comfort and prevent hypothermia [5,30]. A suitable method is to apply heat energy from underneath. So that ambulance personnel may be able to offer safe care for patients, active warming should be procured, for example, through heated mattresses. It may reduce the risk of patients getting chilled from a cold mattress, which in turn may have positive medical consequences, both in the pre-hospital situation and later on in hospital.

This study also showed that the heated mattress was perceived as more pleasant for patients to lie on than the standard mattress. In the earlier discussed Swedish study of 62 patients, 57% deemed the standard mattress to be cold to lie on, while just 3% of patients who got to lie on the heated mattress deemed it to be cold [8]. In another study, participants had a higher back temperature when they had lain on the heated mattress compared with the standard mattress [2]. Cooling the back is an effective way of making a person feel less well or feel uncomfortable in the situation in which they find themselves [31]. In one study conducted on post-operative patients, the best method for regaining warmth was to apply active warming from both above and below [32]. Tsuei and Kearney [15] describe that applying heat through convection and conduction is the best way of warming a patient. It should be obvious even in ambulance care that, when the air temperature drops below zero, a warm ambulance mattress that provides patients with heat is needed. Ambulance care should always strive to care for patients in the best possible way. Optimal care is not always medical; it can also mean just making sure that the patient can lie on something warm and pleasant. If patients are comfortable they will probably be happier and feel better during the care process.

The results also show that personnel thought that patients who lay on the heated mattress were more relaxed. Mild chilling increases muscle tone, making it hard to relax. If the chilling cannot be stopped the patient will begin to shiver. Graham *et al*. [33] demonstrate in their study that shivering is the most common cause of Electro Cardiogram (ECG) disturbances and, of 73 patients with ECG disturbances, 66% were due to shivering. If patients are cold, it can make it difficult to provide good care and medical treatment, because, for example, shivering can give rise to incorrect ECG. Kober *et al*. [34] write that use of a pulse oximeter is an important safety aspect in ambulance care. In this study, of 24 trauma patients who were randomised pre-hospital into two groups, one group were given a battery-powered carbon fibre heating blanket at 42ᵒC, for up to 30–40 minutes. The other group were given the same blanket, but without batteries and unheated. All participants had peripheral vasoconstriction in the fingers before the measurements started and the patients had a transport time of 30 minutes. On arrival at the hospital, patients who had been given the heated blanket had a finger temperature of 34ᵒC, on average, and in the other group the average finger temperature was 25ᵒC. In the group that had been given active warming with the heated blanket, the pulse oximeter functioned during 92% of the transport time and also had a better signal quality from the finger probe. For the group who were given passive warming, the pulse oximeter functioned during 77% of the transport time and had a worse signal quality from the finger probe [34]. These results also show the negative effect of not warming. This can in turn lead to incorrect treatment. A patient not being able to relax or shivering due to being cold is uncomfortable, but it can also on occasion be extremely dangerous for the person affected. To achieve wellbeing and good health for patients, pre-hospital care should be able to prevent chilling. This study shows that, by applying heat from below, patients are deemed by personnel to feel more relaxed on a heated mattress.

Chilling can, thus, be prevented or treated by means of, among other things, blankets and external heating systems as early as in pre-hospital care [35]. However, a lot of resources are usually invested in optimising medical care, while thermal comfort is easily forgotten [36]. Patients starting to shiver makes assessments, conversation and recording of the patient’s medical history more difficult. A heated mattress should be able to prevent patients starting to shiver.

Results also show a tendency for the use of “winter bedding” to reduce somewhat after the heated mattress was introduced, which might be financially advantageous. A patient suffering from mild hypothermia (35–35.9ᵒC) has, among other things, an increased risk of getting an infection [37], with a prolonged care period as a consequence. From a socioeconomic perspective, this increases care costs. Equipping ambulances with heated mattresses entails a one-off cost, but ultimately the patients will be happier and will shiver less. Introducing heated mattresses leads to better care and reduces risks, so that fewer patients will suffer complications, which means a shorter care period. In one study into surgery, those who were not pre-warmed needed a 20% longer hospital stay [38]. Use of polyester blankets alone without an active heat source means patients take longer to warm up again [21]. With the support of Aléx’s [5] PhD thesis, which describes the development of the knowledge surrounding heated mattresses, this constitutes an innovative care development that is both cheap and simple to implement in the care system. Implementation of the heated mattress is a form of improvement work in ambulance care and, therefore, ought to have a major impact. According to Bervick *et al*. [39], it is important to progress from what we know to performing improvement work in care.

### Method discussion

The surveys had study-specific questions that were pilot-tested prior to the measurements to ensure no linguistic misunderstandings arose for the participants. To describe the opinions of ambulance personnel regarding differences between using a heated mattress and a standard ambulance mattress, a quantitative follow-up of the intervention was selected. As early as at the start of the study, many departures and new employments were seen coming among the ambulance personnel. Therefore, coding of the surveys was dropped. If the same participants had responded to both surveys it would have been helpful to do paired t-tests in order to make comparisons at an individual level, instead of at group level, as now. However, the anonymous uncoded data collection at group level probably contributed to a more candid estimate from the informants, since there would otherwise be a risk of bias because the data collection was performed at the same workplace, where two of the authors work on and off. Introducing new procedures can meet with some resistance and for some people it can be hard to change their behaviour, which can be explained by passivity and low motivation [40]. In this study, participation was high, which could indicate that the personnel consider this field of research to be interesting and that there is a need for new products to prevent chilling of patients in ambulance care.

## Conclusion

The ambulance personnel in this study rated the experience of working with the heated mattress as very positive, and proxy rated that it had a good effect on patient comfort. A heated mattress can be recommended for patients in ambulance care, even if more research is needed to receive sufficient evidence.
